# Brief Report: The Vocational and Educational Activities of Transition‐Aged Autistic Youth With Low IQ

**DOI:** 10.1111/jir.70054

**Published:** 2025-09-29

**Authors:** Carly Moser, Ryan Adams, Shuting Zheng, Somer Bishop, Julie Lounds Taylor

**Affiliations:** ^1^ Vanderbilt University Medical Center Nashville Tennessee USA; ^2^ Cincinnati Children's Hospital Medical Center Cincinnati Ohio USA; ^3^ University of California San Francisco California USA

**Keywords:** autism, employment, intellectual disability, postsecondary education, transition to adulthood

## Abstract

**Background:**

Previous research has shown that autistic transition‐aged youth with low IQ experience low rates of integrated employment and postsecondary education (PSE) enrolment. Notably, much of this work was conducted over a decade ago, and the landscape of opportunities has changed since that time. Therefore, the present study described the rate of involvement in vocational and PSE activities among a contemporary sample of autistic youth with low IQ residing in the United States.

**Method:**

Online survey responses were collected from 91 caregivers of autistic youth with low IQ residing in the United States. The survey gathered demographic information and assessed the behavioural functioning of the youth, along with their vocational and educational experiences.

**Results:**

Thirty‐five per cent of autistic youth with low IQ were not involved in any vocational/PSE activity. Approximately 15% of youth were participating in integrated employment (with or without supports), and 5% were enrolled in a range of degree‐ and non‐degree‐seeking PSE programmes. In a follow‐up analysis, we found that youth who were engaged in vocational/PSE activities had higher family incomes, higher daily living skills, and lower rates of borderline‐to‐clinical levels of internalising and externalising behaviours than those who were not engaged in any vocational or PSE activity.

**Conclusions:**

Our findings indicate low participation (~20%) in integrated employment and PSE programmes, despite national efforts to increase these types of activities, and highlight the continued need to remove barriers that prevent engagement in vocational and PSE activities among autistic youth with low IQ.

In the first few years after they exit high school, autistic individuals face substantial barriers to employment and enrolment in postsecondary education (PSE) programmes (Newman et al. 2011; Shattuck et al. 2012). These barriers are especially acute for autistic individuals with low IQ, with some studies finding that under 20% are employed in the community or enrolled in PSE (Billstedt et al. 2011; Taylor and Seltzer 2011). The limited engagement in such activities is concerning, as participation in integrated employment is linked to greater financial security and better psychological health for people with intellectual and developmental disabilities (for a review, see J. Taylor et al. 2022). Moreover, autistic adults and adults with intellectual disability who have participated in degree‐ and non–degree‐seeking PSE demonstrate higher rates of employment and earn more than those with less educational attainment (Moore and Schelling 2015; Whittenburg et al. 2019).

Given the benefits associated with employment and PSE, there has been a surge of research over the past 10 years describing, predicting, and supporting employment for autistic adults, but most of this research does not include autistic adults with low IQ. In fact, much of the work describing employment and PSE activities in autistic adults with low IQ was conducted over a decade ago (for notable exceptions, see Knüppel et al. 2019 and McCauley et al. 2020). Since then, the landscape of employment and PSE opportunities for individuals with intellectual and developmental disabilities has changed. Sheltered work and day programmes historically accounted for a vast majority of vocational opportunities for autistic adults with low IQ (Cederlund et al. 2008; Billstedt et al. 2011; Taylor and Seltzer 2011). However, recent national policy changes in the United States have aimed to increase integrated employment while de‐emphasising sheltered vocational services for individuals with intellectual and developmental disabilities (Wehman et al. 2021). Data collected from a nationwide survey on employment services for individuals with intellectual and developmental disabilities between 1988 and 2021 suggest these changes have indeed resulted in a steady decline in sheltered work and a modest increase in integrated employment services (Zalewska et al. 2023). It is unclear how changes in national funding and regulations in the United States have impacted vocational opportunities for autistic individuals with low IQ who are at the highest risk for unemployment (Taylor and Seltzer 2011; Chiang et al. 2013; McCauley et al. 2020). Further, the number of colleges and universities offering certificate (and sometimes degree‐seeking) PSE programmes targeted to students with intellectual disability has doubled in the past 15 years (Grigal et al. 2018; Think College 2024), though it is unclear to what extent autistic youth with low IQ are participating in these activities. To address this gap in the literature, the present study described the rates of involvement in a range of vocational and educational activities for autistic youth with low IQ in the first few years after leaving high school.

## 1 Method

### 1.1 Participants and Procedures

Participants included 91 caregivers of autistic youth with low IQ (defined as an estimated IQ score of 70 or below), drawn from a larger study of autistic adolescents and young adults with low IQ (J. L. Taylor, Sullivan, et al. 2024). For this analysis, we included youth who had exited the high school system.^1^ Families were recruited from the Simons Simplex Collection registry (SSC; Fischbach and Lord 2010), the Simons Foundation Autism Research for Knowledge (SPARK) research match registry (Feliciano et al. 2018), and various local autism research and clinic registries. Study invitations were sent out to families in the respective registries who had an autistic child in the specified age (15–25 at the time of recruitment) and IQ (70 or below) range. IQ was not directly measured in the current study but was obtained from the existing registries whenever possible or estimated based on caregiver‐report if previously collected IQ data were not available (additional details on the methods used to collect IQ data for each registry are available in the supplemental materials). Caregivers provided electronic consent and completed online surveys about demographics and their child's current behavioural functioning, including daily living skills measured by the Waisman Activities of Daily Living Scale (W‐ADL; Maenner et al. 2013), and internalising and externalising behaviours based on the Child Behavior Checklist 6‐18 (CBCL/6‐18; Achenbach and Rescorla 2001) or the Adult Behavior Checklist (ABCL; Achenbach and Rescorla 2003). Caregivers also provided details about their child's vocational, educational, and social experiences. Data were collected between March 2021 and May 2022. The community advisory committee for the Simons Foundation and the Vanderbilt University Medical Center Institutional Review Board approved all study procedures. Sample characteristics are presented in Table 1.

**TABLE 1:** Sample characteristics.

	M (SD) or *n* (%)
**Youth characteristics**	
Age	23.11 (2.59)
Gender	
Male	71 (78%)
Female	20 (22%)
Race	
Asian	3 (3%)
Black	7 (8%)
Native Hawaiian or other Pacific Islander	1 (1%)
White	75 (82%)
More than one race	5 (6%)
Ethnicity	
Hispanic/Latino	8 (9%)
Not Hispanic/Latino	83 (91%)
Living with caregiver	
Yes	80 (88%)
No	11 (12%)
Daily living skills	18.94 (6.63)
Internalising behaviour	
Normal range	46 (51%)
Borderline to clinical range	44 (49%)
Externalising behaviour	
Normal range	54 (60%)
Borderline to clinical range	36 (40%)
**Caregiver characteristics**	
Age	53.93 (6.81)
Relationship to autistic youth	
Mother	86 (95%)
Other^a^	5 (5%)
Highest level of completed education	
High school diploma or less	27 (30%)
College degree	44 (48%)
Advanced degree	20 (22%)
**Family characteristics**	
Household income	
$20 000 or less	4 (5%)
$20 001–$40 000	13 (16%)
$40 001–$60 000	12 (15%)
$60 001–$80 000	13 (16%)
$80 001–$100 000	11 (13%)
$100 001–$125 000	7 (8%)
$125 001–$150 000	7 (8%)
$150 001–$200 000	5 (6%)
$200 001 or higher	11 (13%)
Classification of residence	
Urban	72 (83%)
Rural	15 (17%)

*Note:* Daily living skills were measured using the total score of the W‐ADL. Internalising and externalising behaviours were measured from the CBCL/ABCL. The classification of residence was obtained by matching each family's zip code to the US Department of Agriculture 2010 rural–urban commuting area codes (USDA Economic Research Service 2023). One participant was missing scores for internalising and externalising behaviour, two were missing scores for daily living skills, eight preferred not to disclose their income, and four were missing rural/urban residence information.

^a^ Four caregivers were fathers and one was a great aunt/guardian.

### 1.2 Measures

#### 1.2.1 Vocational and Educational Activities

The Vocational and Educational Index (VEI; J. L. Taylor, Carlson, et al. 2025) was coded from the survey completed by caregivers that probed for the vocational and educational activities of their autistic youth. The coding scheme contains vocational and educational dimensions with nine categories in each dimension defined by the activity's degree of community integration and support, the outcome (e.g., pay or degree), and whether the youth was participating in the activity for a minimal (10 h or less per week) or more than a minimal amount of time. Two trained research staff coded the VEI, and any discrepancies were reconciled through consensus coding. A third independent research team member coded a random 20% of cases (*n* = 18); agreement with the consensus codes was 94.4% for both the employment and education dimensions. The full list of VEI codes and code descriptions can be found in J. L. Taylor, Carlson, et al. (2025).

### 1.3 Data Analysis

The rates of involvement in vocational and educational activities were examined by calculating the frequency and percentage of participants in each category of the VEI.

## 2 Results

The frequencies and percentages of vocational and educational activities among autistic youth with low IQ are presented in Table 2. Thirty‐two youth (35%) were not involved in any vocational/educational activity. Seventeen youth (19%) participated in activities for 10 h or less a week, of which 41% volunteered a few hours a week. Thus, just over one‐half of youth were engaged in minimal to no vocational/educational activities. Among the 42 (46%) participants who were regularly involved in vocational/educational activities, a little more than one‐half (*n* = 24) participated in sheltered vocational activities, which included day programmes or paid work in settings exclusively for individuals with disabilities.

**TABLE 2:** The frequency of vocational and educational activities among autistic youth with low IQ.

**Regular participation in vocational/educational activities**	42 (46.1%)
Sheltered vocational activities	24 (26.4%)
Paid community employment without supports	5 (5.5%)
Paid community employment with supports	4 (4.4%)
Hybrid vocational activities^a^	3 (3.2%)
Hybrid PSE programme^a^	2 (2.2%)
Volunteering	2 (2.2%)
Degree/credential‐seeking PSE programme	1 (1.1%)
Sheltered PSE programme	1 (1.1%)
**Minimal participation in vocational/educational activities**	17 (18.7%)
Volunteering	7 (7.7%)
Paid community employment with supports	5 (5.5%)
Sheltered vocational activities	4 (4.4%)
Degree/credential‐seeking PSE programme	1 (1.1%)
**No participation in vocational/educational activities**	32 (35.2%)

*Note:* If youth were involved in more than one activity, we present the activity in which they spent the most hours per week. In all cases where multiple activities were endorsed, the second activity was volunteering.

^a^ Hybrid activities are those that have some degree of inclusion with those without disabilities and some degree of participation in segregated settings.

### 2.1 Participation in Integrated Employment

Fourteen autistic youth with low IQ (15%) were employed in the community, with about one‐third (*n* = 5) of these participants working 10 h or less per week. Nine youth who were employed in the community received job supports in the form of a job coach or informal support from family, supervisors, or coworkers. Common duties included stocking shelves, bagging groceries, prepping food, working as a restaurant host, or janitorial work.

### 2.2 Participation in PSE Programmes

Five autistic youth with low IQ (5%) were enrolled in a PSE programme. One participant was taking classes full‐time at a community college, working towards an associate's degree. Another was enrolled in a certification course at a technical school part‐time. Three youth were in PSE programmes targeted to those with intellectual disability; one was taking classes exclusively with other students with disabilities, and two were in hybrid programmes that allow students to take general education classes and courses specifically designed for those with disabilities.

## 3 Follow‐Up Analysis

Given the low rate of engagement in vocational/educational activities, we conducted a follow‐up analysis to see if any characteristics of the sample (see Table 1) differed for those who were involved (i.e., spending any amount of time per week; *n* = 59) versus not involved (i.e., spending no time per week; *n* = 32) in vocational/educational activities. *t* tests were conducted for continuous variables and Fisher's exact tests or chi‐square tests for categorical variables. As shown in Table 3, compared with those who were not engaged in any vocational/educational activity, youth who were participating in activities had higher family incomes, higher daily living skills, and lower rates of borderline‐to‐clinical levels of internalising and externalising behaviours. There was no significant difference between those who were and were not engaged in vocational/educational activities in youth age, gender, race, ethnicity, whether the youth lived with their caregiver, or caregiver age, relationship to youth, education, or whether families lived in an urban (vs. rural) area.

**TABLE 3:** Sample characteristics among youth with low IQ involved in vocational or educational activities compared with those not involved in activities.

	Activities *n* = 59 M (SD) or *n* (%)	No activities *n* = 32 M (SD) or *n* (%)	*t*/*χ* ^2^
**Youth characteristics**			
Age	23.39 (2.40)	22.58 (2.86)	1.36
Gender			0.00
Male	46 (78%)	25 (78%)	
Female	13 (22%)	7 (22%)	
Race			2.79
Asian	2 (3%)	1 (3%)	
Black	6 (10%)	1 (3%)	
Native Hawaiian or other Pacific Islander	1 (2%)	0 (0%)	
White	46 (78%)	29 (91%)	
More than one race	4 (7%)	1 (3%)	
Ethnicity			0.28
Hispanic/Latino	4 (7%)	4 (13%)	
Not Hispanic/Latino	55 (93%)	28 (88%)	
Living with caregiver			0.18
Yes	53 (90%)	27 (84%)	
No	6 (10%)	5 (16%)	
Youth daily living skills	20.20 (6.80)	16.47 (5.61)	2.76**
Youth internalising behaviour			6.65*
Normal range	36 (62%)	10 (31%)	
Borderline to clinical range	22 (38%)	22 (69%)	
Youth externalising behaviour			4.46*
Normal range	40 (69%)	14 (44%)	
Borderline to clinical range	18 (31%)	18 (56%)	
**Caregiver characteristics**			
Age	54.93 (6.79)	52.08 (6.53)	1.96
Relationship to autistic youth			
Mother	54 (92%)	32 (100%)	1.47
Other	5 (8%)	0 (0%)	
Highest level of completed education			4.82
High school diploma or less	15 (25%)	12 (38%)	
College degree	27 (46%)	17 (53%)	
Advanced degree	17 (29%)	3 (9%)	
**Family characteristics**			
Household income^a^	5.34 (2.52)	4.00 (2.13)	2.57*
Classification of residence			1.93
Urban	50 (88%)	22 (73%)	
Rural	7 (12%)	8 (27%)	

*Note:* Chi‐square values are presented even when Fisher's exact test was more appropriate, as the tests yielded the same results. Daily living skills were measured using the total score of the W‐ADL. Internalising and externalising behaviours were measured from the CBCL/ABCL. The classification of residence was obtained by matching each family's zip code to the US Department of Agriculture 2010 rural–urban commuting area codes (USDA Economic Research Service 2023).

^a^ Household income was treated as a continuous variable for the analysis. A household income of ‘4’ corresponds to a range of $60 001–80 000, a ‘5’ corresponds to a range between $80 001–100 000 per year, and a ‘6’ corresponds to a range between $100–001‐125 000 per year.

* *p* < 0.05.

** *p* < 0.01.

## 4 Discussion

The current study found that a little less than half (46%) of transition‐aged autistic youth with low IQ were regularly participating in vocational/educational activities. This is substantially lower than the 92% reported in an earlier study of autistic youth with low IQ of similar age (Taylor and Seltzer 2011). One potential contributor to the low engagement could be the shift in employment and PSE opportunities for those with disabilities over the past decade. There has been a concerted effort to move away from sheltered work opportunities with the goal of increasing engagement in integrated employment (Wehman et al. 2021). Our data suggest that these efforts have been successful in decreasing participation in sheltered activities, as the rate of autistic youth with low IQ participating in segregated settings in this sample (32%) was much lower than previously reported (66%–74%) (Cederlund et al. 2008; Billstedt et al. 2011; Taylor and Seltzer 2011). However, the goal of increasing engagement in integrated employment does not seem to have materialised, as only 15% of autistic youth with low IQ in our sample were involved in community employment, a rate similar to observations from over a decade ago (Billstedt et al. 2011; Taylor and Seltzer 2011; Chiang et al. 2013). Thus, it is essential to continue advocating for policies and investing in programmes that promote successful participation in integrated employment, especially for those with lower cognitive abilities. These efforts are particularly valuable considering the positive impact of integrated employment on financial security and psychological health outcomes for those with intellectual and developmental disabilities (J. Taylor et al. 2022).

Notably, data were collected from March 2021 to May 2022 in the aftermath of the COVID‐19 pandemic. Though many businesses and programmes had reopened by this time, it is possible that extended or permanent closures left some youth without vocational/educational opportunities. This could account for a portion of the sample's disengagement from vocational/PSE activities, but it is unlikely to be the primary explanation. In this sample, there was no difference in engagement (versus disengagement) in vocational/educational activities for those surveyed in 2021 compared with 2022 (*χ* ^2^[1] = 0.64, *p* = 0.424). Further, a study conducted prior to the pandemic—between 2016 and 2018—also found that less than half (43%) of autistic young adults with low IQ were regularly participating in vocational/educational activities (McCauley et al. 2020). Therefore, while the effects of the pandemic cannot be ruled out, it does not appear to be the most likely explanation for low engagement.

Our follow‐up analysis indicated that several youth characteristics, including caregiver‐reported daily living skills and internalising and externalising behaviours, were related to engagement in vocational/educational activities, which aligns with prior work focused on autistic adults with a range of cognitive abilities (Holwerda et al. 2012; Chen et al. 2015). We were unable to disentangle the direction of these associations due to the cross‐sectional nature of the study. One study directly investigating the bidirectional associations between behavioural characteristics and vocational/educational profiles found that engagement in more integrated and independent vocational/educational activities led to subsequent improvements in behavioural characteristics for autistic adults (J. L. Taylor, Smith, et al. 2014). However, there is also evidence that behavioural characteristics are predictive of employment profiles in autistic people with and without intellectual disability (Chiang et al. 2013; Chan et al. 2018; Bury et al. 2024). More work is needed to understand which characteristics might promote employment and which might be a result of employment experiences, but it is possible that treatments aimed at improving daily living skills or ameliorating mental health problems may provide youth with the opportunity to engage more effectively in these settings.

Also consistent with previous research (Chiang et al. 2012, 2013; Shattuck et al. 2012), our results suggest that families with fewer socioeconomic resources—in this case, lower household income—may experience barriers in being able to support their youth's involvement in vocational/educational activities. Due to the inadequacy of adult services, families may need to fund vocational/educational programmes themselves (Anderson and Butt 2018), which can be particularly challenging for those with limited socioeconomic resources. Even for publicly funded activities, constraints around transportation and activities that are provided less than full‐time or have variable schedules may force parents to choose between missing work to support their youth's involvement in programmes or keeping their work hours to avoid losing income—an issue well documented among parents of autistic children (Wallace‐Watkin et al. 2023). Addressing these challenges is essential to promoting equitable access to vocational/educational opportunities for autistic youth.

The study has several limitations, including our relatively small sample size and limited racial/ethnic diversity. Additionally, the varying recruitment sources resulted in inconsistent IQ data, preventing us from further stratifying the sample based on IQ. Although we were unable to delve deeper into the cognitive abilities of youth in this sample, Supporting Information provides greater description of their functional abilities, which may aid in interpreting our results. To capture the vocational/educational experiences of individuals with a range of support needs, we utilised informant‐report but acknowledge it is ideal to capture the perspective of autistic youth when possible.

Other limitations of this analysis point to important directions for future research. First, although the current study was primarily descriptive, our findings can guide future studies in developing hypothesis‐driven research with larger samples to identify the key factors linked to engagement in vocational/educational activities. Secondly, it is unclear the extent to which our findings are unique to autistic youth with low IQ compared with those with low IQ without autism. A report by Bush and Tassé (2017) demonstrated that autistic adults had lower rates of employment than those with idiopathic ID or Down syndrome; the differences between autism and Down syndrome remained even after controlling for key demographic and behavioural variables (including level of intellectual disability). This suggests that there may be vulnerabilities specific to autism that impact access to employment opportunities, though more work is needed to confirm this. Additionally, longitudinal research is needed to understand whether there has been a sustained decrease in vocational/educational participation for autistic youth with low IQ over the past decade and to tease apart the direction of associations between sample characteristics and engagement. The impact of transition planning during high school on participation in vocational/educational activities (which was not considered in the current study) is another important area for future research. Previous studies indicate that autistic students with low IQ are less likely to have required postsecondary goals incorporated into their Individualized Education Program (IEP; Hughes et al. 2023). Further, when these goals are present, they are lower in quality compared with those of their autistic peers with higher IQs (Kraemer et al. 2022). In summary, findings from the study point to a clear and immediate need to promote engagement in vocational/educational activities during the transition to adulthood for autistic youth with low IQ.

## Conflicts of Interest

The authors declare no conflicts of interest.

## Supporting information


**Data S1:** Supporting Information

## Data Availability

The data that support the findings of this study are available on request from the corresponding author. The data are not publicly available due to privacy or ethical restrictions.

## Endnotes

^1^
Nine youth finished or stopped attending high school before the age of 18 (for details surrounding why youth left, see Supporting Information). A sensitivity analysis was conducted for the follow‐up analysis excluding participants under 18 years of age and a similar pattern of findings was observed.
