# Supplementary material for: Brief Report: The Vocational and Educational Activities of Transition‐Aged Autistic Youth With Low IQ
Source: J Intellect Disabil Res. 2025 Sep 29;70(2):218–24. doi: 10.1111/jir.70054 (PMC12757198; doi:10.1111/jir.70054)
Supplement: Supplementary file 1 — Data S1: Supporting Information [file JIR-70-218-s001.docx]

**Supplemental Materials**

**Characteristics of Youth Who Left High School Before 18**

Nine youth exited high school before the age of 18. Although we did not explicitly inquire about the reasons they left, we reviewed relevant survey data to better understand these cases. All nine parents reported that their youth had not received a regular high school diploma. Three parents reported that their youth received a certificate of completion or a modified diploma, while five others mentioned that their youth left high school without any diploma. One parent reported that their youth was homeschooled, had less than a high school education, and had earned a homeschool diploma.

**Methods for Collecting IQ**

We relied on existing databases and registries to recruit the study sample. All participants were identified by having an IQ score of 70 or below; however, the methods used to collect and report IQ scores varied across the registries. Sixteen families in the current study were recruited through the Simons Simplex Collection registry (SSC; Fischbach & Lord, 2010), 72 from the Simons Foundation Autism Research for Knowledge research match registry (SPARK; Feliciano et al., 2018), and 3 through local autism research registries. For the SSC and local autism research registries, IQ was assessed at the time of initial enrollment using a variety of standardized assessments, including the Mullen Scales of Early Learning, the Differential Ability Scales-II, the Wechsler Intelligence Scale for Children-IV or the Wechsler Abbreviated Scale of Intelligence. The majority of IQ scores for SPARK participants were obtained from parent-reported IQ ranges, though a minority (*n*=2) had previous records of clinical evaluations available.

**Supplemental Characteristics of the Sample: Daily Living Skills**

To better illustrate the functional support needs of our sample, we categorized participants according to their level of independence in basic daily living skills using items from the Waisman Activities of Daily Living Scale (W-ADL; Maenner et al., 2013). The W-ADL probes caregivers to rate their child’s level of independence across 17 different activities of daily living, such as toileting, doing laundry, and managing daily finances. Parents rate whether their child can independently perform the task (‘2’), does the task with help (‘1’), or does not do the task at all (‘0’). We examined what proportion of the sample was independent (i.e., caregivers endorsed a code of 2) in each of the following basic daily living skills: ‘drinking from a cup,’ ‘eating from a plate,’ ‘washing/bathing,’ ‘grooming, brushing teeth, combing or brushing hair,’ ‘toileting,’ and ‘dressing and undressing.’

Thirty-nine percent of our sample (*n*=35) were independent in all of these basic daily living skills and 61% (*n*=54) were not. A higher percentage of autistic youth with low IQ who engaged in vocational/educational activities were independent in basic daily living skills (*n*=28; 47%) compared to youth who were not engaged in any vocational/educational activity (*n*=7; 23%; *χ*^2^(1) = 3.89, *p* = .049), mirroring findings when we examined the full range of W-ADL scores.
